# Cohort Profile: A nationwide Swedish register-based cohort of older adults on housing, residential relocation, and healthy ageing (Register RELOC-AGE)

**DOI:** 10.1093/ije/dyaf115

**Published:** 2025-07-07

**Authors:** Giedre Gefenaite, Jonas Björk, Susanne Iwarsson

**Affiliations:** Faculty of Medicine, Department of Health Sciences, Lund University, Lund, Sweden; Division of Occupational and Environmental Medicine, Lund University, Lund, Sweden; Clinical Studies Sweden, Forum South, Skåne University Hospital, Lund, Sweden; Faculty of Medicine, Department of Health Sciences, Lund University, Lund, Sweden

**Keywords:** living environment, moving trajectories, population-based studies, longitudinal, health

Key Features

- Register RELOC-AGE was established to study housing and relocation in relation to active and healthy ageing and provides a unique opportunity for longitudinal population-based studies focusing on person–environment relations.
- Register RELOC-AGE is a general population register-based cohort in Sweden with yearly data available between 1987 and 2021, extended every third year.
- Register RELOC-AGE includes >3 million index participants aged ≥55 years and their partners irrespective of their age, and is regularly updated, totalling ∼5.1 million.
- The data come from 13 national Swedish registers and 1 regional database, including individual-level information on health, social and home care, medication prescriptions, demographic and socioeconomic characteristics, as well as data on >2.7 million housing units (e.g. type, tenure, size), relocations over 30 years, and perceived outdoor environment characteristics.
- Proposals for possible collaboration and data access should be sent to the Lund University Research Data Office (request@researchdata.lu.se) as well as Assoc. Prof. Giedre Gefenaite [giedre.gefenaite@med.lu.se] or Prof. Susanne Iwarsson (Principal Investigator) [susanne.iwarsson@med.lu.se].

## Why was the cohort set up?

Providing housing and living environments that promote active and healthy ageing is a critical challenge, especially in societies experiencing the fastest rate of population ageing. Yet, there is a lack of evidence from population-based quantitative studies on how to create age-friendly living environments, particularly when considering the complex contexts in which people age [1, 2].

One of the few comprehensive literature reviews available on the topic indicated that factors potentially influencing housing decisions among older adults are many, very diverse, and have rarely been studied in depth with the aim of ‘embracing the transdisciplinary complexity’ [3]. To date, cross-sectional and small-scale longitudinal studies have shown that housing accessibility, operationalized as a person–environment interaction [4, 5], is associated with health-related outcomes among older adults and those ageing with underlying neurological illnesses [6–9]. These studies suggested that housing and health are linked through various pathways that may be dependent on the studied population. Yet, longitudinal studies of scale that could provide stronger causal evidence about housing and health links and the underlying pathways are scarce.

When it comes to residential relocation histories, studies that extend beyond frail populations, single moves, or short time frames are rare. A variety of different relocations and their trajectories can be identified, such as moves between different types and tenures of housing, to moves farther apart geographically or based on certain area-level characteristics, such as deprivation, population density, or outdoor environment qualities ([10–12] and Mtutu *et al.*, under revision). However, a better understanding of residential relocation histories and their links to health along the process of ageing is necessary to enable evidence-based housing planning, practices, and policies.

Since the onset of the COVID-19 pandemic, housing and living environments have received substantially more attention, largely due to pandemic control measures such as social isolation. At the beginning of the pandemic, 90% of the population reported avoiding leaving their homes [13]. A study conducted in Brazil showed that residing in multifamily dwellings and row houses, as compared with detached houses, was associated with a decrease in physical activity and an increase in sedentary behaviours among adults aged 60–80 years with hypertension [14]. A Swedish register-based study of people aged ≥55 years found that, during the first year of the pandemic, living in long-term care facilities was associated with a 22‐fold increase in the odds of dying due to COVID-19 [15]. Such findings highlight the home as an important health determinant in later life because it influences health behaviours and exposes individuals to risks related to different living environments. To date, longitudinal studies on the links between indoor and outdoor living environment characteristics and their effects on communicable and non-communicable diseases in later life remain scarce.

To address the lack of evidence regarding the links between housing, relocations, and health along the process of ageing, we for the first time linked comprehensive person and housing (including neighbourhood) data available from population registers in Sweden. We developed an inter/nationally unique data structure that allowed the study of hitherto understudied questions in the Register RELOC-AGE cohort, which is representative of Sweden, over a period of >30 years. The cohort was set up to explore: (i) time trends when it comes to housing types and tenures; (ii) how housing aspects and relocations affect future health outcomes; (iii) short- and long-term effects of health events on housing choices and relocation trajectories; and (iv) how housing aspects are related to direct and indirect COVID-19 health outcomes, and how they compare to the effects during the A(H1N1)pdm09 and other influenza epidemics. We will investigate how the results based on these questions are affected by demographic, socioeconomic, and health characteristics, and their changes. We will also investigate the contexts in which people age by examining socio-demographic characteristics, adverse health events, and/or loss of their partners. As Register RELOC-AGE is part of a large research programme (see below), we will also delve into active and healthy ageing measures based on population register data (e.g. based on multi- and comorbidity, frailty indices, and mortality).

Register RELOC-AGE is part of the RELOC-AGE research programme and is linked to Prospective RELOC-AGE [16], which is a longitudinal survey following people aged ≥55 years with an intention to relocate (*N* = 1964). The programme is situated at the Department of Health Sciences, Lund University and implemented in the context of the inter- and transdisciplinary Centre for Ageing and Supportive Environments (CASE) and Proactive Ageing profile area. CASE bridges research at four Lund University faculties. As part the university-wide profile area, Register RELOC-AGE aims to contribute to the development of strategies to promote activity and participation along the process of ageing.

Register RELOC-AGE was supported by grants from the Swedish Research Council (SRC) (grant nos. 2019–00996; 2022–00521) and the Swedish Research Council for Sustainable Development (FORMAS) (grant nos. 2020–02881, 2019–01916). In addition, the Faculty of Medicine, Lund University research allocation funding and an annual donation from 2005 onwards from the Ribbingska Foundation in Lund are used to support the work. Continuous efforts are made to obtain further funding to maintain the cohort beyond 2027. In addition, the principal investigator has made contact to explore possibilities to include the cohort in an existing national infrastructure for register-based research on ageing. This infrastructure has long-term extensive funding from the SRC and mechanisms for funding and outreach to researchers with an interest in making use of existing data sources are in place.

## Who is in the cohort?

Register RELOC-AGE is a population-based cohort from the Swedish Total Population Register (TPR) [17] of ∼1.8 million persons born in 1908–31 and still alive in 1987 and 3.3 million individuals born in 1932–61 as they turned ≥55 years during 1987–2016. The study started in 1987, as this was when the National Patient Register (NPR) [18] became nationwide. To address the contexts in which the ≥55-year-old participants age, we have identified and/or additionally included their spouses/registered partners during the study period, irrespective of their age, resulting in a total sample of 5.1 million. There were 3 021 282 individuals in the Register RELOC-AGE index cohort alive on 31 December 2016 (the year in which the initial enrolment into the cohort closed). For descriptive characteristics of the cohort, see Table 1. The majority lived in single- and multifamily housing, with ∼3% staying in residential care facilities.

**Table 1.** Descriptive characteristics of the Register RELOC-AGE ≥55-year-old index cohort alive on 31 December 2016 (*N* = 3 021 282)

	Housing type				
Characteristic	Residential care	Single-family	Multifamily	Other	Total
*N* (%)	98 251 (3.3)	1 700 319 (56.3)	1 185 870 (39.3)	36 842 (1.2)	3 021 282 (100.0)
Age group (years) [*n* (%)]					
55–64	6995 (7.1)	674 490 (39.7)	415 728 (35.1)	12 035 (32.7)	1 109 248 (36.7)
65–74	12 916 (13.1)	653 512 (38.4)	399 637 (33.7)	11 474 (31.1)	1 077 539 (35.7)
75–84	27 103 (27.6)	292 151 (17.2)	255 793 (21.6)	8420 (22.9)	583 467 (19.3)
85+	51 237 (52.1)	80 166 (4.7)	114 712 (9.7)	4913 (13.3)	251 028 (8.3)
Sex [*n* (%)]					
Men	33 605 (34.2)	879 948 (51.8)	506 163 (42.7)	16 127 (43.8)	1 435 843 (47.5)
Women	64 646 (65.8)	820 371 (48.2)	679 707 (57.3)	20 715 (56.2)	1 585 439 (52.5)
Civil status [*n* (%)]					
Married/reg. partner	15 536 (15.8)	1 122 714 (66.0)	452 768 (38.2)	14 756 (40.1)	1 605 774 (53.1)
Other	82 715 (84.2)	577 605 (34.0)	733 102 (61.8)	22 086 (59.9)	1 415 508 (46.9)
Education level [*n* (%)]					
Primary (≤9 years of compulsory education)	51 456 (53.4)	439 498 (25.9)	351 714 (29.8)	10 869 (29.7)	853 537 (28.4)
Secondary	32 482 (33.7)	747 649 (44.1)	502 349 (42.6)	15 226 (41.6)	1 297 706 (43.1)
Tertiary (professional/university (college))	12 397 (12.9)	509 750 (30.0)	325 124 (27.6)	10 537 (28.8)	857 808 (28.5)
Income quintiles, lowest to highest [*n* (%)]					
1st	54 034 (55.0)	223 851 (13.2)	311 541 (26.3)	9703 (26.3)	599 129 (19.8)
2nd	26 162 (26.6)	291 170 (17.1)	279 796 (23.6)	8242 (22.4)	605 370 (20.0)
3rd	8683 (8.8)	363 091 (21.4)	228 038 (19.2)	6442 (17.5)	606 254 (20.1)
4th	3332 (3.4)	406 513 (23.9)	190 991 (16.1)	5704 (15.5)	606 540 (20.1)
5th	6040 (6.1)	415 694 (24.4)	175 504 (14.8)	6751 (18.3)	603 989 (20.0)
Home care service [*n* (%)]					
Yes	84 647 (86.2)	91 708 (5.4)	157 708 (13.3)	6657 (18.1)	340 720 (11.3)
No	13 604 (13.8)	1 608 611 (94.6)	1 028 162 (86.7)	30 185 (81.9)	2 680 562 (88.7)
Nordic origin [*n* (%)]					
Yes	93 583 (95.2)	1 615 150 (95.0)	1 016 416 (85.7)	33 594 (91.2)	2 758 743 (91.3)
No	4668 (4.8)	85 169 (5.0)	169 454 (14.3)	3248 (8.8)	262 539 (8.7)
Household size [*n* (%)]					
Single person	NA	466 006 (27.4)	670 279 (56.5)	20 575 (55.8)	1 242 250 (41.1)
Two-person	NA	1 024 067 (60.2)	440 582 (37.2)	13 980 (37.9)	1 491 054 (49.4)
Three-person or more	NA	210 246 (12.4)	75 009 (6.3)	2287 (6.2)	287 978 (9.5)
Children <18 years old in the household [*n* (%)]					
Yes	NA	51 540 (3.0)	25 498 (2.2)	693 (1.9)	77 824 (2.6)
No	NA	1 648 779 (97.0)	1 160 372 (97.8)	36 149 (98.1)	2 943 458 (97.4)
Housing tenure [*n* (%)]					
Rented	NA	47 510 (2.8)	619 238 (52.2)	30 471 (82.7)	777 663 (25.8)
Tenant-owned	NA	59 905 (3.5)	566 126 (47.7)	6369 (17.3)	637 423 (21.1)
Owner-occupied	NA	1 592 538 (93.7)	408 (0.0)	0 (0.0)	1 599 651 (53.1)
Year of building, in tertiles [*n* (%)]					
<1958	8417 (14.1)	639 355 (37.7)	336 162 (28.4)	11 430 (36.4)	995 364 (33.5)
1958–75	16 858 (28.1)	523 023 (30.9)	479 935 (40.6)	9043 (28.8)	1 028 859 (34.7)
>1975	34 629 (57.8)	531 393 (31.4)	367 184 (31.0)	10 935 (34.8)	944 141 (31.8)
Hospitalizations during past three years (2013–15) [*n* (%)]					
Yes	60 704 (61.8)	420 332 (24.7)	351 882 (29.7)	11 898 (32.3)	844 816 (28.0)
No	37 547 (38.2)	1 279 987 (75.3)	833 988 (70.3)	24 944 (67.7)	2 176 466 (72.0)

**Figure 1.**
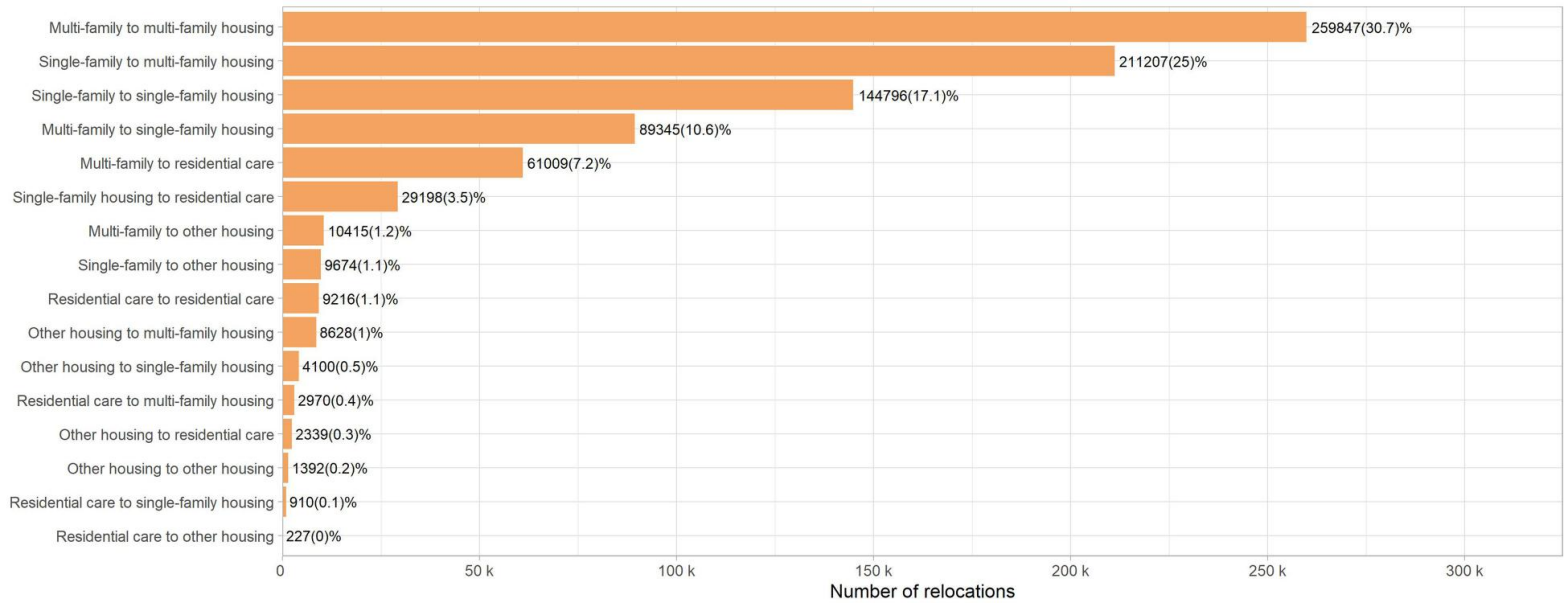
Relocations between different housing types in the Register RELOC-AGE ≥55-year-old index cohort (*N* = 720 766).

In addition, Register RELOC-AGE includes data about 720 766 residential relocations of the aged ≥55 years index cohort between 2012 and 2020 (identification of earlier relocations is also possible) and detailed data about each of the 2.7 million housing units in which the participants lived during the period. For different types of relocations, see Figure 1.

The linkage of the register data is based on personal identification numbers (PINs) and housing unit numbers and implemented by Statistics Sweden. The PIN is a unique identifier used across societal sectors (e.g. health, social care, financial) and enables data linkage across various registers. In Sweden, a PIN is assigned to everyone registered in the TPR [19]. Persons with protected identity (0.01% persons of any age in 2014 [17], which had nearly doubled by 2021) were not included. Before linking the TPR to other registers, individuals with duplicate PINs were excluded (0.1%).

Register RELOC-AGE was approved by the Swedish Ethical Review Authority (EPM; nos. 2020–01369, 2021–01124, 2024–02460-02), allowing additional data to be obtained until 31 March 2027 or extended upon further request. Further EPM amendments will be sought as applicable. Following the principles routinely applied for register-based research in Sweden, no informed consent for the participants was required. Instead, opt-out messages for participants were posted on the Lund University website.

## How often have they been followed up?

The cohort was followed continuously during 1987–2021 and covered ∼97% of the ≥55-year-old Swedish population in 2016. Currently, the data are available for nearly 35 years and >120 million person-years. Most of the data are available based on when an event of interest (e.g. prescription, diagnosis) occurred. Some data are available as a monthly or yearly indication of a status change (e.g. starting to receive care upon disability or certain age) or frequency (e.g. services per month, year). We plan the next data update during 2025.

## What has been measured?

**Figure 2.**
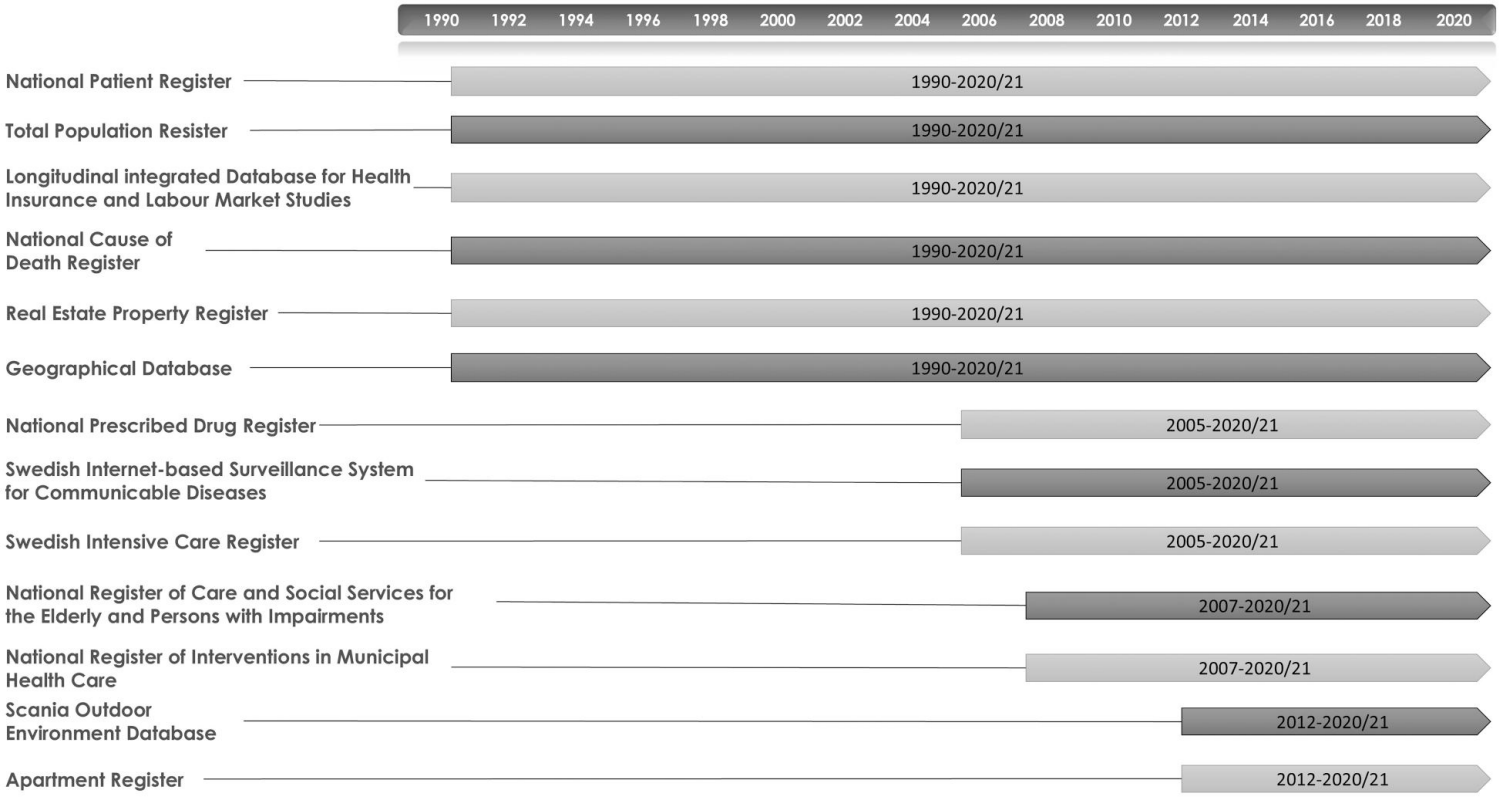
An overview of the 13 Swedish registers and regional databases included in Register RELOC-AGE and the available data timelines.

For each participant, data from the TPR, NPR, and the National Cause of Death Register (NCDR) [20] since 1987 and the Real Estate Property Register (REPR) [21] and Longitudinal integrated database for health insurance and labour market studies (LISA) [22] since 1990 were linked. The NPR is used to extract data on hospitalizations and outpatient visits on ageing-related outcomes (e.g. falls and fractures, stroke), chronic medical conditions (e.g. cardiovascular diseases, diabetes), and mental disorders (e.g. depression, anxiety). The participants have also been linked with the National Prescribed Drug Register (NPDR) [23] since 2005, the National Register of Care and Social Services for the Elderly and Persons with Impairments (NRCSS) [24] since 2007, the National Register of Interventions in Municipal Health Care (NRIMHC) [25] since 2007, and the Apartment Register (AR) [26] since 2012. To account for the consequences of the COVID-19 pandemic and compare them with those of influenza epidemics and pandemics, we linked data on intensive care admissions due to COVID-19 from the Swedish Intensive Care Registry (SIR; www.icuregswe.org) for 2020 and laboratory-based diagnoses of COVID-19 and influenza from the Swedish internet-based surveillance system for communicable diseases (SmiNet) [27] from 2009, with the latter including the infectious diseases that are mandatory to report. Additionally, the participants living in Scania (Skåne) in southern Sweden were linked to the Scania Outdoor Environment Database (ScOut) [28], which is a unique data source allowing close outdoor living environment effects to be accounted for. Not least, Scania includes densely populated cities, mid-sized towns, as well as sparsely populated rural areas, and is socioeconomically diverse, allowing rather complex studies of the environment and health dynamics. Other environmental data can be linked to Register RELOC-AGE through geocodes to be able to take a variety of outdoor exposures into account. For an overview of the register data and timelines, see Figure 2.

## What has it found?

Within the available data structure, we explored the housing trajectories between 2012 and 2020 among the ≥55-year-olds and found that 14% relocated at least once. Among the movers, eight major relocation trajectories were identified, of which three were relocating within the same and five transitioning to different housing types and tenures (Mtutu *et al.*, under revision). Several demographic, socioeconomic, and health characteristics at baseline predicted which trajectories individuals followed. For example, as compared with men, women were 33% more likely to move from single-family housing ownership to multifamily housing renting. Higher education consistently reduced downsizing moves, such as to rental housing. Mental health problems increased, while physical health problems reduced the chances of relocation. Another study showed that living in areas with the most perceived greenness (i.e. a combined area-level score of nature qualities such as serene, natural, diverse, and cohesive [28]) was associated with lower COVID-19-related mortality and hospitalization, yet the precision after the adjustment for potential confounders was lacking (Bhebhe *et al.*, under revision). In yet another study, compared with living in single-family housing, living in residential care facilities during the COVID-19 pandemic was associated with a few times higher incidence of laboratory-confirmed SARS-CoV-2, which increased later in the pandemic (Gefenaite *et al.*, in submission). Several other studies looking into the associations and the mechanisms between different housing and outdoor environment characteristics and health are currently ongoing.

All publications will appear on https://portal.research.lu.se/en/projects/reloc-age-how-do-housing-choices-and-relocation-matter-for-active.

## What are the main strengths and weaknesses?

The Register RELOC-AGE data structure is inter/nationally unique. There is a close to complete coverage of the ageing population in Sweden from ≥55 years of age, the follow-up length is nearly 35 years, and >120 million person-years, with detailed individual and housing data that are linked from a number of sources. As part of the RELOC-AGE research programme, Register RELOC-AGE benefits from and complements other parts of the programme, and is therefore in a strong position to generate novel knowledge regarding the effects of housing and relocations on active and healthy ageing.

Including study participants already at the age of 55 years allows us to capture person–environment dynamics at target from an earlier phase in life than is usually performed in research on ageing and housing (including neighbourhoods). To be able to address the social context in which the participants are ageing, another notable strength is including demographic, socioeconomic, and health data about their spouses/registered partners (irrespective of age). Living environment characteristics obtained through population-based registers is still an underused alternative to generate new knowledge about the actual links between housing, relocation, and active and healthy ageing and trajectories over time. Linking population-based registers provides a hitherto never used opportunity to investigate housing and health associations in later life longitudinally. Register RELOC-AGE makes it possible to conduct statistically powered longitudinal studies across a broad span of older adults, assessing the role of various housing and outdoor environment characteristics on a range of health conditions. In addition, linking specific cohorts of older adults to Register RELOC-AGE, such as the subsample of 1964 individuals from the Prospective RELOC-AGE [16], is currently in progress.

Linking TPR, NPR, NCDR, LISA, SmiNet, and SIR provides data of high quality. The remaining registries included pose some challenges due to shorter follow-up and/or data quality, to be taken into careful consideration during the design and analysis phases, as well as when interpreting and reporting the results. Yet, there are benefits to including these registers. For example, while data in the REPR are detailed and generally of high quality, one known limitation is that relocations to special housing such as residential care facilities are not well captured because those who moved there may not have updated their residential addresses. Through linkage to the NRCSS, capturing social care interventions for older adults and people with impairments, we can to some extent counteract this limitation. In addition, linking the REPR to the AR adds more detailed information about the dwellings, such as the size, number of rooms, building year, etc. (since 2012). NPDR data make it possible to use detailed information on health conditions and primary healthcare use since 2005. While still under development and not of optimal quality, registers with data on municipal healthcare and social services, such as the NRCSS and NRIMHC, are essential for studies addressing active and healthy ageing, and will be used as complementary data sources. Strategies to decrease the risks of bias (e.g. limiting the analysis to fewer years or municipalities) and those of known and unknown quality limitations and strengths are continuously considered. Last but not least, ScOut provides area-aggregated measures on perceived outdoor environment characteristics for Scania [28], enabling us to address the effects of close outdoor living environments on active and healthy ageing in a subsample of ≥300 000 participants within Register RELOC-AGE.

## Can I get hold of the data? Where can I find out more?

Proposals for possible collaboration should be sent to the Lund University Research Data Office (request@researchdata.lu.se) or directly to Assoc. Prof. Gefenaite [giedre.gefenaite@med.lu.se] and Prof. Iwarsson, principal investigator [susanne.iwarsson@med.lu.se]. Each case will be considered in terms of research scope and the applicable ethical regulations of the EPM and the General Data Protection Regulation are met, aiming to facilitate research collaboration with local, national, and international partners.

## Ethics approval

Register RELOC-AGE was approved by the EPM (nos. 2020–01369, 2021–01124, 2024–02460-02), allowing additional data to be obtained until 31 March 2027 or extended upon further request. Further EPM amendments will be sought as applicable.

## Data Availability

Sharing of individual-level data within Register RELOC-AGE requires a new ethical permission and a confidentiality review by the data host (Lund University).
